# Prevalence and factors associated with non-medical needs among adult cancer patients attending a tertiary care center in Kerala, India. A cross-sectional study

**DOI:** 10.3389/fpubh.2025.1734144

**Published:** 2026-01-13

**Authors:** Jeby Jose Olickal, Kizhakke Neelamana Sandhya, Sreelekshmi Sreedharan, Shivani Rengan, Kavumpurathu Raman Thankappan, Keechilat Pavithran

**Affiliations:** 1 Department of Public Health, Amrita Institute of Medical Sciences, Amrita Vishwa Vidyapeetham, Kochi, Kerala, India; 2 Department of Medical Oncology, Amrita Institute of Medical Sciences, Amrita Vishwa Vidyapeetham, Kochi, Kerala, India

**Keywords:** cancer patients, cross-sectional study, health disparities, India, Kerala, non-medical needs, oncology, social determinants of health

## Abstract

**Introduction:**

Social determinants of health (SDH) significantly influence cancer risk, treatment adherence, and prognosis; however, research assessing multiple SDH factors, particularly in the Indian context, remains limited. This study aims to determine prevalence of non-medical (social) needs and identify the factors associated with at least one such need among adult cancer patients attending a tertiary care center in Kochi, Kerala, India.

**Methods:**

This cross-sectional study was conducted at a private tertiary cancer care center. A total of 362 adult cancer patients (aged ≥ 18 years, 64.3% female) attending the oncology outpatient department were consecutively recruited. Data were collected through structured interviews using the WellRx questionnaire to identify non-medical (social) needs. Associations between sociodemographic and clinical factors and the presence of any non-medical need were assessed and adjusted prevalence ratios (APR) were estimated with Poisson regression.

**Results:**

Overall, 220 patients (60.8%; 95% CI: 55.8%−65.5%) patients reported at least one non-medical need. The most commonly reported needs were employment or regular income (*n* = 145; 40.1%), transportation (*n* = 83; 22.9%), and payment of utility bills (*n* = 74; 20.4%). In multivariable analysis, patients aged < 50 years (APR 1.37; 95% CI: 1.01–1.84; *p* = 0.042) and 51–59 years (APR 1.42; 95% CI: 1.09–1.85; *p* = 0.008) were more likely to report at least one non-medical need compared to those aged ≥70 years. Similarly, patients with primary education (APR 1.50; 95% CI: 1.20–1.86; *p* < 0.001), secondary education (APR 1.28; 95% CI: 1.04–1.57; *p* = 0.020), and those belonging to below poverty line (BPL) households (APR 1.24; 95% CI: 1.02–1.50; *p* = 0.028) had a significantly higher prevalence of non-medical needs.

**Conclusion:**

More than half of adult cancer patients reported at least one non-medical (social) need, highlighting the importance of routine social needs screening in oncology care. Targeted support is warranted for patients who are unemployed, have lower educational attainment, belonging to BPL households, or are aged 51–59 years.

## Introduction

Cancer is a major global public health concern, ranking as the second leading cause of death worldwide. In 2020, an estimated 19.3 million new cancer cases and nearly 10 million cancer-related deaths were reported, with projections indicating a rise to 28.4 million cases annually by 2040 (1). The most commonly diagnosed cancers include breast, lung, colorectal, prostate, and stomach cancer, with significant variations across regions and populations (2). Low- and middle-income countries (LMICs) bear a disproportionate burden, with limited healthcare access and late-stage diagnoses contributing to high mortality rates (3). In India, cancer incidence is also on the rise, with approximately 1.39 million new cases and nearly 850,000 deaths in 2020 (4). The most prevalent cancers in the country include breast, oral, cervical, lung, and stomach cancers, which account for a substantial portion of the disease burden (5). Regional disparities, healthcare accessibility issues, and financial constraints contribute to delayed diagnosis and suboptimal treatment, leading to poor survival outcomes (6).

The increasing burden of cancer emphasize the importance of addressing disparities in healthcare access, treatment adherence, and patient outcomes. Social determinants of health (SDH) play a crucial role in shaping cancer risk, progression, and survival by influencing socioeconomic status, healthcare accessibility, lifestyle behaviors, social support, and psychological well-being (7). Socioeconomic inequalities often lead to delayed diagnoses and limited treatment options, particularly among disadvantaged populations (8). Geographic barriers, lack of health insurance, and financial constraints further hinder access to timely and appropriate care (9). Additionally, factors such as tobacco use, alcohol consumption, poor diet, and sedentary lifestyles contribute significantly to the rising cancer burden (10). The psychological impact of cancer, including stress, anxiety, and depression, also affects treatment adherence and quality of life (11). Understanding the influence of these determinants is essential for developing effective interventions aimed at reducing disparities and improving cancer care.

Despite increasing recognition of SDH in cancer care, there remains a gap in comprehensive research assessing multiple SDH factors simultaneously, particularly in the Indian context. Most studies focus on isolated determinants such as income or healthcare access, without considering the interconnected nature of these factors and their cumulative impact on patient outcomes (12). This study aims to determine prevalence of non-medical (social) needs and identify the factors associated with at least one such need among adult cancer patients attending a tertiary care center in Kochi, Kerala, India.

## Methods

### Study design

This study was a hospital based cross sectional study.

### Study setting

The study was conducted at, a private tertiary cancer care center in Kerala, India. This center serves as a major referral center for oncology patients from various regions of Kerala, providing comprehensive cancer diagnosis, treatment, and supportive care while catering to patients from diverse socioeconomic backgrounds.

### Study population

The study included adult cancer patients aged 18 years or older with histologically confirmed malignancies who were undergoing treatment at the tertiary care center and provided informed consent to participate. Patients with cognitive impairments that affected their ability to respond to the questionnaire and those unwilling to participate were excluded from the study.

### Study duration

The study was conducted from August-September 2025.

### Sample size

Assuming a prevalence of at least one non-medical (social) need among cancer patients of 46%, as reported by Page-Reeves et al. (13), and considering a 95% confidence level with 5% absolute precision, the required sample size was calculated to be 362 using OpenEpi (version 3.0).

### Sampling technique

Patients were consecutively recruited from the oncology outpatient department (OPD) until the required sample size of 362 was achieved. All eligible patients attending the OPD during the study period and meeting the inclusion criteria were invited to participate.

### Study procedure

Patients meeting the inclusion criteria were identified during their hospital visits and approached for participation. After obtaining informed consent, structured interviews were conducted to assess socioeconomic status, healthcare access, social support, lifestyle behaviors, and psychological well-being. Additionally, medical record reviews were performed to extract details on time from symptom onset to diagnosis, cancer stage at diagnosis, and treatment adherence rates.

### Data collection

Patients were consecutively recruited from the oncology outpatient department. Data were collected through structured face-to-face interviews and medical record reviews by trained research personnel.

The interview schedule included the WellRx questionnaire, which was used to identify non-medical or social needs across multiple domains. The WellRx questionnaire has been previously developed and used to assess patients' non-medical (social) needs (13). Sociodemographic information; including age, sex, religion, education, occupation, type of family, marital status, place of residence, and socioeconomic status (ration card type); was recorded. As per the ration card poorer households are given the below poverty line (BPL) cards.

Clinical information; including type and stage of cancer, presence of chronic illness, family history of cancer, and treatment details (chemotherapy, surgery, and radiotherapy recommendations); was extracted from medical records.

### Study tools

The WellRx questionnaire was used as the primary tool to assess social determinants of health (SDH) among cancer patients. It is a validated instrument designed to measure non-medical factors influencing health outcomes, including financial difficulties, housing instability, food insecurity, healthcare access barriers, and psychosocial stressors. Permission to use the WellRx questionnaire was obtained from the developers. The questionnaire consists of 11 Yes/No questions, with each affirmative response (“Yes”) indicating the presence of a social risk factor.

The WellRx questionnaire covered five key domains:

1. Socioeconomic status – assessed income, employment, and education to evaluate financial stability and its impact on healthcare access.
2. Healthcare access – evaluated transportation barriers, insurance coverage, and financial constraints affecting medical expenses.
3. Social support – examined household safety, exposure to domestic violence, and the availability of family and community assistance in managing health-related needs.
4. Lifestyle factors – assessed food security, substance use, and childcare needs to identify areas requiring intervention.
5. Psychological well-being – measured stress, safety concerns, and emotional distress, recognizing their influence on mental health and treatment adherence (13).

### Independent and outcome variables

The outcome variable in this study was the presence of any non-medical need, defined as reporting at least one unmet social need on the WellRx questionnaire.

The independent variables included sociodemographic factors such as age, gender, religion, marital status, type of family, place of residence, educational qualification, occupation, and socioeconomic status (based on ration card type), and clinical characteristics including history of chronic illness, family history of cancer, and stage at diagnosis.

### Ethical considerations

The study protocol was reviewed and approved by the institute ethics committee of Amrita School of Medicine (Approval Number: ECASM-AIMS-2024-609). Written informed consent was obtained from the patients.

### Statistical analysis

Data were entered in Microsoft Excel sheets and analyzed using STATA version 14. Categorical variables, including sex, residence, education, occupation, socioeconomic status, and clinical characteristics, were expressed as frequencies and percentages. The proportion of patients reporting at least one non-medical need on the WellRx questionnaire was calculated with corresponding 95% confidence intervals (CI). Associations between sociodemographic and clinical characteristics and the presence of any non-medical need were examined using log-binomial regression and unadjusted prevalence ratios (UPR) with 95% CIs were calculated. Variables with *p* < 0.2 in the unadjusted analysis were entered into a multivariable Poisson regression model with robust variance to obtain adjusted prevalence ratios (APR) and 95% CI. A *p*-value < 0.05 was considered statistically significant.

## Results

As shown in Table 1, most patients were aged 60–69 years (*n* = 113; 31.2%), female (*n* = 233; 64.4%), and Hindu (*n* = 268; 74.0%). Half were unemployed (*n* = 184; 50.83%), and the majority had tertiary education (*n* = 230; 63.5%). Most held an Above Poverty Line (APL) ration card (*n* = 318; 87.9%), belonged to nuclear families (*n* = 272; 75.1%), were married (*n* = 298; 82.3%), and resided in urban areas (*n* = 189; 52.2%). Chronic illness was reported by 158 (43.7%), and family history of cancer by 101 (28.1%). The most common stage at diagnosis was stage 3 (*n* = 137; 38.0%), and the majority were advised chemotherapy (*n* = 318; 87.9%).

**Table 1:** Sociodemographic and clinical characteristics of the study patients (*N* = 362).

**Variable**	**Category**	***n***	**%**
Age (years)	<50	81	22.4
	51–59	99	27.4
	60–69	113	31.2
	≥70	69	19.1
Gender	Female	233	64.4
	Male	129	35.6
Religion	Christian	62	17.1
	Hindu	268	74.0
	Muslim	32	8.8
Educational qualification	Primary	72	19.9
	Secondary	60	16.6
	Tertiary	230	63.5
Type of ration card	Above Poverty Line	318	87.9
	Below Poverty Line	44	12.2
Type of family	Joint Family	90	24.9
	Nuclear Family	272	75.1
Marital status	Married	298	82.3
	Single	64	17.7
Place of residence	Rural	173	47.8
	Urban	189	52.2
Any history of chronic illness	No	204	56.4
	Yes	158	43.7
Any history of cancer in the family	No	259	71.9
	Yes	101	28.1
Stage at diagnosis	1	19	5.3
	2	115	31.9
	3	137	38.0
	4	90	24.9
Chemotherapy advised	No	44	12.2
	Yes	318	87.9
Surgery recommended	No	198	54.7
	Yes	164	45.3
RT advised after CT/surgery	No	235	64.9
	Yes	127	35.1

**Figure 1:**
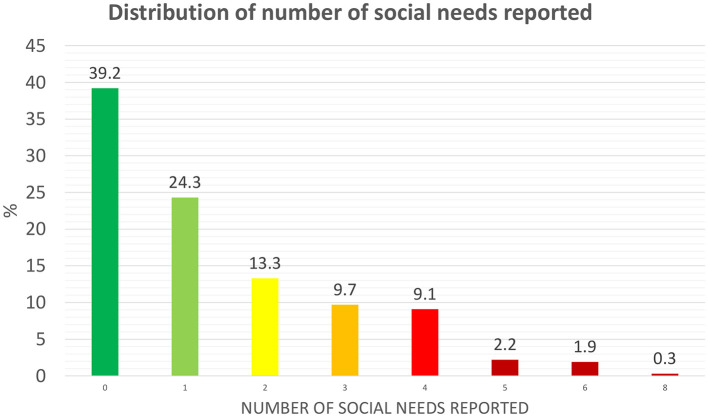
Distribution of number of social needs reported.

As presented in Table 2, the most frequently reported needs were employment or regular income (*n* = 145; 40.1%), transportation (*n* = 83; 22.9%), and payment of utility bills (*n* = 74; 20.4%). Overall, 220 patients (60.8%, 95% CI 55.5%−65.8%) reported at least one non-medical need, while 142 (39.2%) reported none (Figure 1).

**Table 2:** Non-medical needs identified in the study population (*N* = 362).

**WellRx questionnaire**	**Response Yes *n* (%)**	**%**
In the past 2 months, did you or others you live with eat smaller meals or skip meals because you didn't have money for food?	17	4.7
Are you homeless or worried that you might be in the future?	40	11.1
Do you have trouble paying for your utilities (gas, electricity, phone)?	74	20.4
Do you have trouble finding or paying for a ride?	83	22.9
Do you need day care, or better day care, for your kids?	14	3.9
Are you unemployed or without regular income?	145	40.1
Do you need help finding a better job?	40	11.1
Do you need help getting more education?	15	4.1
Are you concerned about someone in your home using drugs or alcohol?	21	5.8
Do you feel unsafe in your daily life?	59	16.3
Is anyone in your home threatening or abusing you?	3	0.8

Sociodemographic and clinical factors associated with any non-medical need are presented in Table 3. Patients aged < 50 years (APR 1.37; 95% CI: 1.01–1.84; *p* = 0.042) and 51–59 years (APR 1.42; 95% CI: 1.09–1.85; *p* = 0.008), those with primary education (APR 1.50; 95% CI: 1.20–1.86; *p* < 0.001) or secondary education (APR 1.28; 95% CI: 1.04–1.57; *p* = 0.020), and patients from BPL households (APR 1.24; 95% CI: 1.02–1.50; *p* = 0.028) were significantly more likely to report at least one non-medical need.

**Table 3:** Sociodemographic and clinical factors associated with any non-medical need: results from univariate and multivariable regression analysis (*N* = 362).

**Category**	***n***	**Any need *n* (%)**	**No need *n* (%)**	**UPR (95% CI)**	***p*-value**	**APR (95% CI)**	***p*-value**
**Age (years)**							
<50	81	51 (63.0)	30 (37.0)	1.24 (0.93–1.65)	0.139	1.37 (1.01–1.84)	0.042
51–59	99	73 (73.7)	26 (26.3)	1.45 (1.12–1.89)	0.005	1.42 (1.09–1.85)	0.008
60–69	113	61 (54.0)	52 (46.0)	1.06 (0.80–1.42)	0.672	1.10 (0.83–1.45)	0.502
≥70	69	35 (50.7)	34 (49.3)	1	-	1	-
**Gender**							
Female	233	150 (64.4)	83 (35.6)	1.19 (0.99–1.43)	0.070	0.127	0.127
Male	129	70 (54.3)	59 (45.7)	1	–	–	–
**Religion**							
Christian	62	35 (56.5)	27 (43.6)	1	–	1	–
Hindu	268	162 (60.5)	106 (39.6)	1.07 (0.84–1.36)	0.575	1.01 (0.80–1.27)	0.943
Muslim	32	23 (71.9)	9 (28.1)	1.27 (0.94–1.73)	0.124	1.12 (0.82–1.51)	0.480
**Educational qualification**							
Primary	72	58 (80.6)	14 (19.4)	1.53 (1.30–1.81)	0.000	1.50 (1.20–1.86)	<0.001
Secondary	60	41 (68.3)	19 (31.7)	1.30 (1.05–1.60)	0.015	1.28 (1.04–1.57)	0.020
Tertiary	230	121 (52.6)	109 (47.4)	1	–	1	–
**Type of ration card**							
APL	318	181 (56.9)	137 (43.1)	1	–	1	–
BPL	44	39 (88.6)	5 (11.4)	1.56 (1.35–1.80)	0.000	1.24 (1.02–1.50)	0.028
**Type of family**							
Joint	90	57 (63.3)	33 (36.7)	1.06 (0.88–1.27)	0.558	–	–
Nuclear	272	163 (59.9)	109 (40.1)	1	–	–	–
**Marital status**							
Married	298	176 (59.1)	122 (40.9)	1	–	1	–
Single	64	44 (68.8)	20 (31.3)	1.16 (0.96–1.41)	0.118	1.02 (0.84–1.24)	0.837
**Place of residence**							
Rural	173	116 (67.1)	57 (33.0)	1.22 (1.03–1.44)	0.020	1.03 (0.86–1.24)	0.727
Urban	189	104 (55.0)	85 (45.0)	1	–	1	–
**Any history of chronic illness**							
No	204	119 (58.3)	85 (41.7)	1	–	–	–
Yes	158	101 (63.9)	57 (36.1)	1.10 (0.93–1.29)	0.276	–	–
**Any history of cancer in the family**							
No	259	153 (59.1)	106 (40.9)	1	–	–	–
Yes	101	65 (64.4)	36 (35.6)	1.09 (0.91–1.30)	0.343	–	–
**Stage at diagnosis**							
1	19	12 (63.3)	7 (36.8)	1.14 (0.78–1.66)	0.498	1.00 (0.70–1.43)	0.999
2	115	75 (65.2)	40 (34.8)	1.18 (0.96–1.44)	0.114	1.16 (0.96–1.41)	0.127
3	137	76 (55.5)	61 (44.5)	1	–	1	–
4	90	56 (62.2)	34 (37.8)	1.12 (0.90–1.40)	0.307	1.13 (0.92–1.39)	0.259
**Chemotherapy advised**							
No	44	28 (63.6)	16 (36.4)	1.05 (0.83–1.34)	0.668	–	–
Yes	318	192 (60.4)	126 (39.6)	1	–	–	–
**Surgery recommended**							
No	198	124 (62.6)	74 (37.4)	1.07 (0.90–1.27)	0.430	–	–
Yes	164	96 (58.5)	68 (41.5)	1	–	–	–
**Radiotherapy advised after CT/surgery**							
No	235	150 (63.8)	85 (36.2)	1.16 (0.96–1.39)	0.118	1.13 (0.95–1.35)	0.163
Yes	127	70 (55.1)	57 (44.9)	1	–	1	–

APL, above poverty line; BPL, below poverty line, UPR, unadjusted prevalence ratio from log-binomial models. APR, adjusted prevalence ratio from Poisson regression with robust variance (adjusted for age, occupation, education, ration card, and place of residence).

## Discussion

This cross-sectional study revealed that 60.8% of adult cancer patients in Kerala reported at least one non-medical or social need, underscoring the role of social determinants of health in influencing cancer outcomes. The most frequently cited needs employment (40.1%), transportation (22.9%), and to pay utility bills (20.4%) reflect the multidimensional financial and logistical strain associated with cancer care. These findings align with the work of Zafar et al. (8), who described “financial toxicity” among insured cancer patients in the United States, demonstrating that out-of-pocket expenses and work loss contribute substantially to distress. Similarly, Mallath et al. (6) observed that up to 60.0% of Indian patients experience catastrophic health expenditures during treatment. Hence, despite Kerala's relatively advanced healthcare system, economic pressures continue to act as barriers to equitable cancer care access.

Our prevalence of 60.8% for any non-medical need aligns with recent Indian evidence showing widespread unmet supportive care requirements. Gupta et al. (14) reported that all 256 cancer patients in rural North India had palliative care needs spanning biopsychosocial domains, while 56.6% had moderate-to-severe physical and social symptoms such as pain, transportation barriers, and social discrimination. Likewise, Muralidharan et al. (15) found that 89% of caregivers in Pune reported financial stress, emotional exhaustion, and time burden, especially among spouses balancing employment with caregiving. These parallels reinforce that social hardship persists across settings, irrespective of regional development indicators, signifying structural weaknesses in cancer care systems. Qualitative research supports our findings that unmet needs often extend beyond financial aspects to include communication and psychosocial domains. Chawak et al. (16) reported that Indian cancer patients frequently experienced unsatisfactory communication with healthcare providers and inadequate emotional support from peers, leading to unmet expectations from their support networks. This aligns with our patients' high prevalence of socioemotional challenges. Together, these studies underscore that comprehensive cancer care in India requires greater attention to psychosocial and informational dimensions, not merely medical treatment.

Globally, disparities in supportive care needs reflect systemic inequities. Williams et al. (17) explained that social determinants such as neighborhood environment, education, and healthcare access: create reinforcing cycles that sustain global cancer disparities. Chan et al. (18) in a multinational survey of supportive care specialists, found that LMIC respondents prioritized issues like transportation, drug affordability, and guideline implementation; mirroring the main barriers identified in our cohort. Conversely, in high-income countries (HICs) like Australia, Molassiotis et al. (19) demonstrated markedly lower unmet psychosocial needs, attributable to integrated welfare systems and coordinated oncology services. Thus, our findings reflect a persistent divide between HICs and LMICs in meeting cancer patients' non-medical needs.

Despite such contrasts, even HICs face challenges in integrating psychosocial care. Signorelli et al. (20) reported that while 39% of centers in HICs routinely performed distress screening, only 25% globally offered structured psychosocial interventions, citing barriers like limited workforce and insufficient policy support. These global insights contextualize our findings within a broader continuum of care inadequacies and highlight that equity in oncology extends beyond clinical advancements to encompass systemic social protection mechanisms.

In this study younger patients exhibited a significantly higher prevalence of unmet non-medical needs. Patients aged below 50 years and those between 51–59 years were more likely to report at least one such need. This trend highlights that working-age patients face the dual burden of illness and financial disruption due to employment loss or reduced productivity. Comparable findings have been reported by Haier et al. (9), who emphasized that productivity loss during cancer treatment contributes to downward economic mobility and unmet supportive care needs, particularly in LMICs. Moreover, the National Cancer Registry Programme reported a rising trend of cancer in younger adults (4), further amplifying the urgency to address these economic challenges during active disease management.

In our study, educational attainment was another key determinant, with patients having primary education and secondary education demonstrating greater unmet needs compared to those with tertiary education. This observation aligns with the WHO framework on social determinants of health, which underscores the role of education in empowering patients to navigate healthcare systems effectively (7). Similar patterns have been described by Islami et al. (10), who found that low educational levels were associated with poorer health literacy and increased delays in treatment initiation. In our setting, limited education may hinder awareness of financial aid programs, insurance benefits, or rehabilitation services, compounding the likelihood of unmet social needs. Targeted health education and literacy-sensitive interventions are therefore essential to mitigate these inequalities.

Economic status also played a substantial role, as patients BPL households in this study had higher likelihood of reporting at least one non-medical need. The proportion of participants belonging to BPL households in our study was considerably lower than that reported in the general population of Kerala, suggesting a possible underrepresentation of economically disadvantaged groups. This lower representation is likely because the study was conducted in a private tertiary cancer care center, where poorer patients are less likely to seek treatment compared to public sector hospitals. This observation is further supported by the Economic Review 2024 of the Government of Kerala, which reports a substantially higher proportion of BPL households in the state than that observed in our study (21), underscoring the socioeconomic selectivity of patients accessing private cancer care. The economic gradient observed here is consistent with evidence from the National Cancer Registry, which indicates that out-of-pocket payments account for up to 70% of cancer-related expenditures in India (4). Similarly, Zafar et al. (8) and Islami et al. (10) observed that lower-income groups, even in high-income countries, face greater levels of treatment-related financial hardship. Our data reaffirm that poverty not only influences access to care but also sustains long-term psychosocial and material vulnerabilities throughout the cancer care continuum. Expanding financial protection schemes and improving BPL eligibility for cancer-specific assistance could significantly alleviate such disparities. The findings from our study suggest the urgent need to institutionalize screening for social determinants of health within oncology practice. The WellRx tool, used in this study, has previously demonstrated feasibility for identifying and addressing social needs within primary care settings (13). Integrating such screening into oncology clinics could guide targeted interventions: financial counseling, travel subsidies, and social welfare linkage programs: particularly for vulnerable groups such as younger, low-educated, and BPL patients.

The study's strengths include the use of a validated WellRx tool, ensuring reliable and generalizable findings. However, as the study was conducted in a single private tertiary hospital in urban Kochi, the findings may not reflect the situation in public hospitals or rural areas, where unmet needs could be higher due to greater socioeconomic constraints. In addition, data collection was limited to a short, fixed period (August–September 2025), which may have introduced selection bias, as patients presenting during this timeframe may not fully represent the broader cancer patient population across different seasons or periods. The reliance on self-reported data may have introduced social desirability bias, as patients could underreport sensitive issues such as financial distress or family problems; although interviewer training helped minimize this, it cannot be fully eliminated. Patients with advanced-stage disease or hospitalization were excluded to ensure uniform data collection, possibly leading to an underestimation of true unmet needs in the sickest populations. Finally, given the cross-sectional design, causal relationships between sociodemographic factors and unmet needs cannot be inferred.

## Conclusion

More than half of the patients reported unmet needs, with economic, educational, and age-related disparities strongly influencing vulnerability. Targeted support is particularly warranted for patients who are unemployed, have lower educational attainment, belong to BPL households, or are aged 51–59 years. Systematic screening and targeted social interventions are essential to achieve equitable, patient-centered oncology outcomes in India and similar resource-limited contexts. Expanding public-sector coverage and developing low-cost, community-based psychosocial support models can help close the disparity gap between LMICs and HICs.

## Data Availability

The raw data supporting the conclusions of this article will be made available by the authors, without undue reservation.
